# Fourth Primary Malignant Tumor in a Patient with Possible Li-Fraumeni Syndrome: Synchronous Diagnosis of Postirradiation Sarcoma, Cutaneous Relapse of a Previous Soft Tissue Sarcoma, and Lung Adenocarcinoma

**DOI:** 10.1155/2014/597207

**Published:** 2014-11-18

**Authors:** Feridun Yumrukçal, Yalin Dirik, Arda Çinar, Levent Eralp

**Affiliations:** ^1^Orthopaedics and Traumatology, Memorial Sisli Hospital, Piyalepasa Bulv., Sisli, 34385 Istanbul, Turkey; ^2^Orthopaedics and Traumatology, Istanbul University School of Medicine, Turgut Özal Millet Caddesi, Çapa Tip Fakültesi, Fatih, 34098 Istanbul, Turkey

## Abstract

We present a 46-year-old female patient who is diagnosed with synchronous postirradiation sarcoma, cutaneous relapse of a previous soft tissue sarcoma, and lung adenocarcinoma. More than one malignant tumor at the same time with an accompanying relapse of a previous malignant tumor is a rare entity. A relatively young patient diagnosed with adenocarcinoma of the urethra before age 40, which is an unusual tumor for that age, later three more different malignant tumors being diagnosed, two of which are synchronous, causes the suspicion of Li-Fraumeni syndrome.

## 1. Introduction

Li-Fraumeni syndrome is a rare genetic condition and increases the risk of developing more than one cancer including breast cancer, soft tissue sarcomas, osteosarcoma, and brain tumors [1, 2]. Clinical diagnosis of classic Li-Fraumeni syndrome is a proband with sarcoma diagnosed before age 45, a first degree relative with any cancer before age 45, and a first or second degree relative with any cancer before age 45 years or a sarcoma at any age [2].

Generally accepted criteria to diagnose a postirradiation sarcoma (PIS) include treatment with therapeutic irradiation at least two years prior to development of a sarcoma, a sarcoma arising within the field of previous therapeutic irradiation, and different histologic features between the sarcoma and the primary tumor that required radiotherapy [3, 4].

With this report we aimed to present a 46-year-old female patient who is diagnosed with a possible Li-Fraumeni syndrome with four primary malignant tumors, one of which is a postirradiation sarcoma with simultaneous relapse of the previous sarcoma within the same anatomic compartment.

## 2. Case Report

We present a 46-year-old female patient. The patient was first diagnosed with urethra adenocarcinoma 8 years ago. Total resection of the urinary bladder with lymphatic dissection was performed followed by combined chemotherapy and radiotherapy. One and a half-year after the first operation the patient was reoperated on for metastasis of the urethral adenocarcinoma and vulvectomy with left inguinal radical lymph node dissection was performed. The pathology report was in compliance with the first report of the urethral adenocarcinoma. Postoperatively, the patient received 54 Gy/30 fraction radiotherapy. At the end of the same year the patient suffered a mass in her right thigh that caused a suspicion of another metastasis. Tru-cut biopsy was performed and pathology specimens revealed malignant mesenchymal tumor. 28 Gy/8 fraction preoperative radiotherapy was applied due to close neighbourhood of the tumor to the femoral artery and the tumor being larger than 5 centimeters in diameter. Following preoperative radiotherapy, wide resection of the tumor was performed. The pathology reports showed a high grade pleomorphic sarcoma. The oncology consultation did not recommend postoperative chemotherapy, but 20 Gy/10 fraction adjuvant radiotherapy was applied.

**Figure 1:**
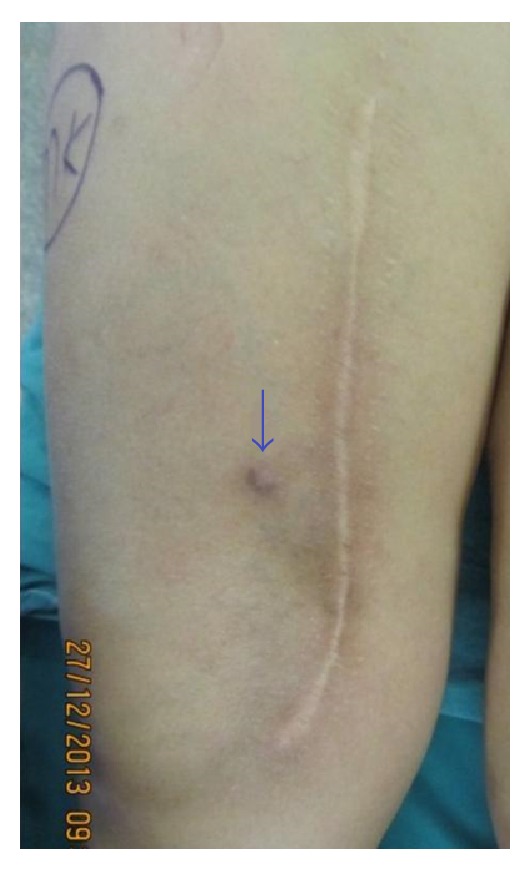
Clinical photograph shows cutaneous relapse.

**Figure 2:**
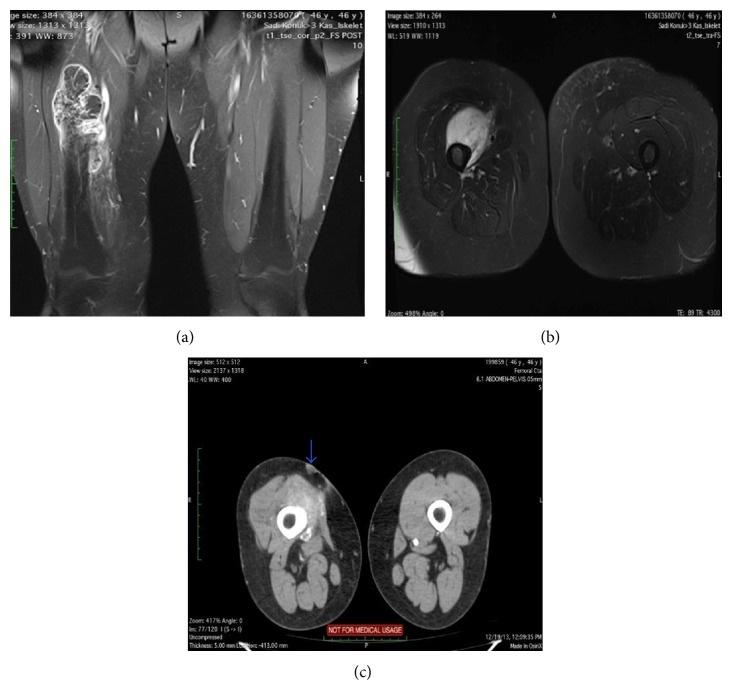
(a) MRI demonstrating tumoral lesion (coronal). (b) MRI demonstrating tumoral lesion (axial). (c) MRI demonstrating cutaneous relapse.

**Figure 3:**
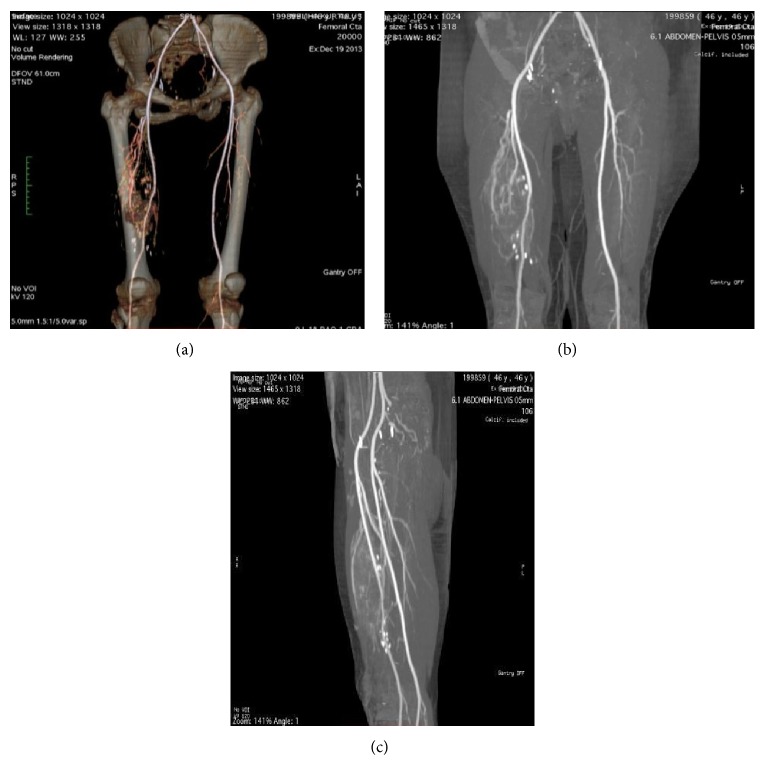
(a) Three-dimensional CT angiography. (b) Coronal plane CT angiography. (c) Sagittal plane CT angiography.

For the next three years the patient was regularly followed and no evidence of local or systemic relapse was present. The patient quit the routine control examinations and could not be followed until 4 months ago when MRI evaluation was performed for a mass in her left thigh. A subcutaneous lesion was detected in the left thigh with typical features of a lipoma. In addition, a large mass in the right femur was coincidentally observed with a satellite cutaneous nodule close to the previous incision scar (Figures 1 and 2). Tru-cut biopsy and PET scan were performed to grade the new lesion. The tru-cut biopsy has reported a low grade periosteal chondral tumor. An apical lesion of the right lung which was thought to be a metastasis was also present. MRI and CT angiographic evaluation showed the soft tissue component of the tumor enclosing the bone lesion 180 degrees medially and also involving a 10 cm segment of the femoral artery and vein (Figure 3).

**Figure 4:**
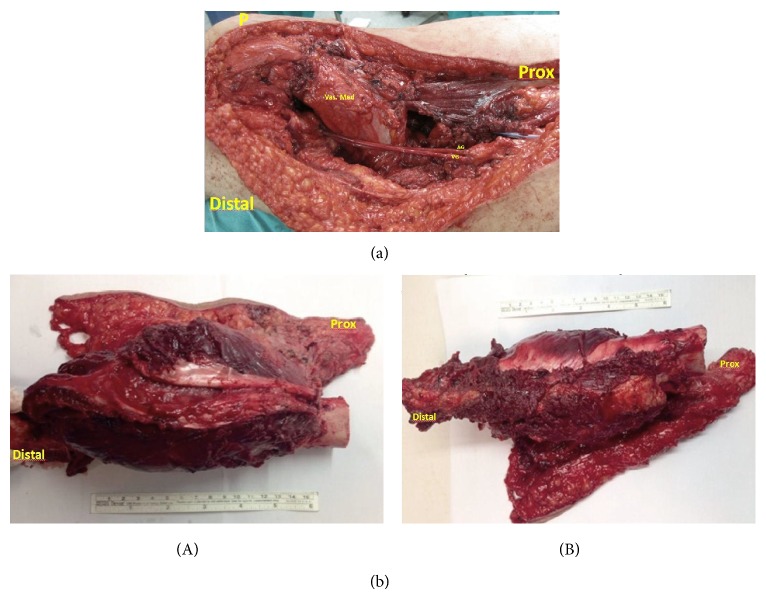
(a) Intraoperative picture demonstrating soft tissue coverage of massive allograft and reconstructed femoral artery and vein using saphenous vein graft (P: patella, Prox: proximal, Vas. Med: vastus medialis muscle, AG: arterial graft, and VG: vein graft). (b) (A, B) The resectate.

**Figure 5:**
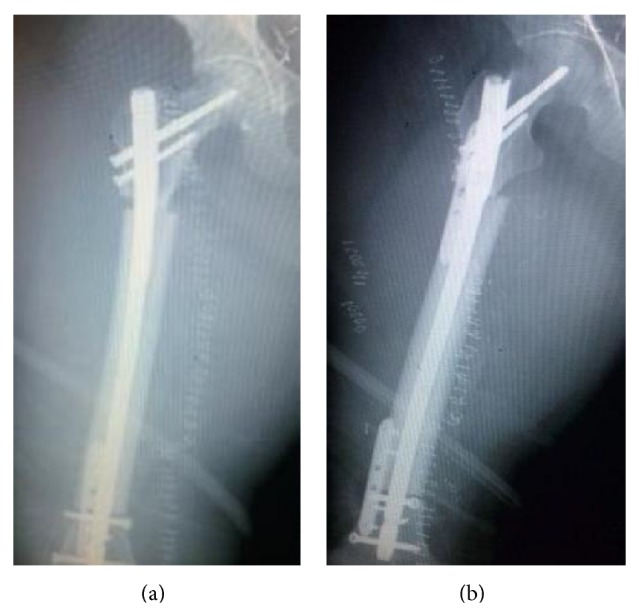
((a), (b)) Early postoperative AP and lateral X-rays.

The patient was reoperated on. Wide resection for the metastatic nodule of the skin was performed first and the frozen pathology reported it as a malignant mesenchymal tumor. The tumor of the right femur was also removed with wide resection including the involved 10 cm segment of the femoral artery and vein in the Hunter canal (Figure 4). Frozen pathology results reported clean margins and the lesion being possibly periosteal chondroblastic osteosarcoma. The femoral artery and vein were reconstructed using saphenous vein from the left leg. Saphenous vein of the right leg was not used in order not to disturb both venous drainage systems of the same leg and to avoid a possible venous insufficiency postoperatively. The 22 cm long bony defect was reconstructed using a massive allograft of femur and plate-screw fixation of the proximal and distal osteotomy sites was performed following locked intramedullary nailing (Figure 5). The docking sites were grafted with 10 cc of demineralized bone matrix each. Pathologic evaluation revealed the mass of the right femur to be a juxtacortical chondroblastic osteosarcoma and the soft tissue mass anterior to it to be an indifferentiated pleomorphic sarcoma, the latter being in compliance with a relapse of the pleomorphic mesenchymal tumor that was resected in 2008. Pathologic evaluation of the resected dumbbell-shaped lesion of the left thigh revealed angiolipoma.

**Figure 6:**
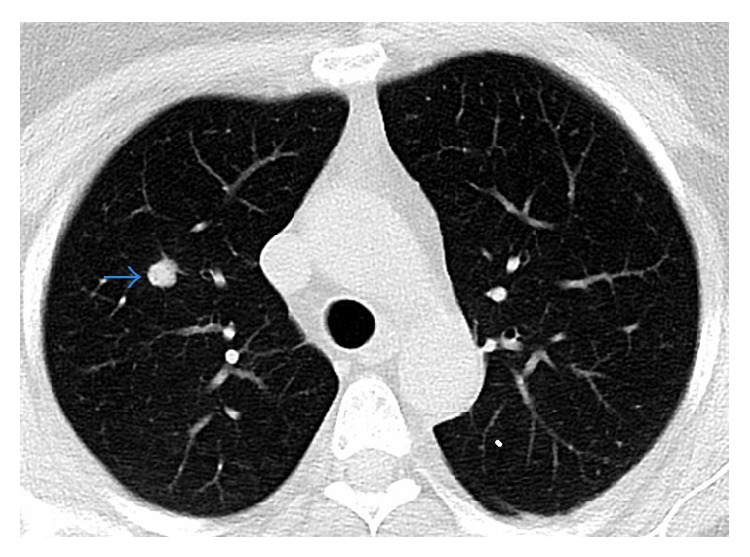
Thorax CT showing lesion in the right lung.

Throughout the routine radiologic evaluations and PET scan before surgery, a lesion in the right lung was also detected and was reported as a metastatic lesion (Figure 6). Three weeks after the last surgery, thoracoscopy assisted wedge resection of the metastatic lesion was planned. At this time, the frozen biopsy displayed a primary adenocarcinoma of the upper lobe of the right lung and the patient underwent a lobectomy.

## 3. Discussion

Li-Fraumeni syndrome is a rare cancer predisposition disorder that is associated with germline mutations of the p53 tumor suppressor gene. The three clinical criteria for typical Li-Fraumeni are a proband with sarcoma diagnosed before age 45, a first degree relative with any cancer before age 45, and a first or second degree relative with any cancer before age 45 years or a sarcoma at any age. Patients with Li-Fraumeni syndrome are more prone to having more than one primary tumor and are also more prone to have postirradiation sarcomas due to TP53 gene mutation [5–7]. In a study, it was observed that 50% of Li-Fraumeni syndrome family members developed a second sarcoma in the radiotherapy field [5].

The fact that the tumor must arise in the region of a prior irradiation, having a latency period of at least two years, and proof that the sarcoma is histologically different from the radiated primary lesion are the criteria of postradiation sarcoma modified by Laskin et al. [4, 8]. A high rate of TP53 gene mutation was observed in radiation-induced sarcomas compared to sporadic ones with studies examining mutations in specific genes using polymerase chain reaction followed by direct sequencing (88% versus 20% and 58% versus 16.8% for two different series) [5]. Although the dose of fractioned radiation exposure is also important for the development of PIS, there is evidence that sarcomas can also be induced by acute lower doses of radiation (<5 Gy) [5].

Our patient had four different malignancies at the age of 46 including two different carcinomas and two different sarcomas. Her son also died because of metastatic complications of osteosarcoma. The first lesion that she was operated on for was urethral adenocarcinoma at the age of 37 which is a carcinoma type that is usually seen after 70 years of age and is atypical for her age [9]. The lesion in her thigh was diagnosed juxtacortical chondroblastic osteosarcoma that is an uncommon osteosarcoma subtype [10] and was accepted as a PIS that developed only three years after the irradiation of the field. Two years after the irradiation is needed to be accepted as a PIS, but the mean time for a PIS to develop is more than 10 years [8]. With this kind of a history, although the third criterion for diagnosis is not met for clinical diagnosis, the patient shows a possible Li-Fraumeni syndrome with TP53 gene mutation [10].

In a study, chemotherapy followed by surgical resection is proposed for PIS and according to the same study the patients who benefited from complete surgical resection had the best prognosis [3]. Although chemotherapy is rarely proposed or considered as the main treatment of PIS while osteosarcomas are particularly more sensitive to chemotherapy, we treated our patient with wide resection of the tumor followed by chemotherapy. Many authors recommend treating PIS in the same way as sporadic sarcomas [3]. The patient in our case previously received radiotherapy of high doses and developed two different sarcomas and two different carcinomas. The possibility of our patient having Li-Fraumeni syndrome, who developed a PIS, directed us to chemotherapy while in both situations there is a possibility of TP53 gene mutation increasing the risk of a new PIS formation.

Reconstruction of diaphyseal bone defects after tumor resection can be performed using different biologic or nonbiologic materials including diaphyseal endoprostheses, allografts, bone transport, and vascularized autograft [11–13].

Although nonbiologic materials provide immediate reconstruction, high implant failure rates that cause need for additional operations are likely. A reconstruction using an endoprosthesis might be a better alternative for patients with poor survival expectation as these allow immediate weight bearing [11, 14]. A biologic reconstruction is a better choice for a younger patient with long-term survival. Use of a vascularized bone autograft is a technically demanding procedure and the transported fibula is unlikely to enlarge sufficiently in width especially for femoral replacement and should be kept as an alternative for young children who are less likely to experience complications such as stress fracture and malunion [11, 14]. With our case, while the femoral artery and vein were also involved in the tumor, it was even more challenging to perform vascularized bone grafting. Induced membrane technique is a relatively new technique that could also be performed, but a two-stage surgery was not preferred [11].

We performed massive allograft reconstruction using an intramedullary nail. The advantage of this kind of a reconstruction is that it allows mechanical and biologic reconstruction during the same procedure [12–14]. The complications include fracture, nonunion, and infection. During short- and mid-term followups the patient did not experience infection. We grafted the docking site to decrease the risk of nonunion and implant failure due to nonunion.

A patient with an atypical cancer for his/her age and with a first degree relative who had a sarcoma history, as was the situation in our case, should be examined carefully and the possibility of Li-Fraumeni syndrome should be kept in mind in order not to increase the risk of PIS due to treatment with high doses of neoadjuvant and adjuvant radiotherapy. In such a situation, chemotherapy added to surgical resection could also be a choice of treatment.

## References

[1] Mai P. L., Malkin D., Garber J. E., Schiffman J. D., Weitzel J. N., Strong L. C., Wyss O., Locke L., Means V., Achatz M. I., Hainaut P., Frebourg T., Evans D. G., Bleiker E., Patenaude A., Schneider K., Wilfond B., Peters J. A., Hwang P. M., Ford J., Tabori U., Ognjanovic S., Dennis P. A., Wentzensen I. M., Greene M. H., Fraumeni J. F., Savage S. A. (2012). Li-Fraumeni syndrome: report of a clinical research workshop and creation of a research consortium. *Cancer Genetics*.

[2] Malkin D. (2011). Li-fraumeni syndrome. *Genes & Cancer*.

[3] Des Guetz G., Chapelier A., Mosseri V., Dorval T., Asselain B., Pouillart P. (2009). Postirradiation sarcoma: clinicopathologic features and role of chemotherapy in the treatment strategy. *Sarcoma*.

[4] Orosz Z., Rohonyi B., Luksander A., Szántó J. (2000). Pleomorphic liposarcoma of a young woman following radiotherapy for epithelioid sarcoma. *Pathology & Oncology Research*.

[5] Berrington de Gonzalez A., Kutsenko A., Rajaraman P. (2012). Sarcoma risk after radiation exposure. *Clinical Sarcoma Research*.

[6] Heymann S., Delaloge S., Rahal A. (2010). Radio-induced malignancies after breast cancer postoperative radiotherapy in patients with Li-Fraumeni syndrome. *Radiation Oncology*.

[7] Li F. P., Fraumeni J. F., Mulvihill J. J., Blattner W. A., Dreyfus M. G., Tucker M. A., Miller R. W. (1988). A cancer family syndrome in twenty-four kindreds. *Cancer Research*.

[8] Laskin W. B., Silverman T. A., Enzinger F. M. (1988). Postradiation soft tissue sarcomas: an analysis of 53 cases. *Cancer*.

[9] Gakis G., Witjes J. A., Compérat E., Cowan N. C., De Santis M., Lebret T., Ribal M. J., Sherif A. M. (2013). EAU guidelines on primary urethral carcinoma. *European Urology*.

[10] Hauben E. I., Arends J., Vandenbroucke J. P., van Asperen C. J., van Marck E., Hogendoorn P. C. (2003). Multiple primary malignancies in osteosarcoma patients. Incidence and predictive value of osteosarcoma subtype for cancer syndromes related with osteosarcoma. *European Journal of Human Genetics*.

[11] Biau D. J., Pannier S., Masquelet A. C., Glorion C. (2009). Case report: reconstruction of a 16-cm diaphyseal defect after Ewing's resection in a child. *Clinical Orthopaedics and Related Research*.

[12] Muscolo D. L., Ayerza M. A., Aponte-Tinao L., Ranalletta M., Abalo E. (2004). Intercalary femur and tibia segmental allografts provide an acceptable alternative in reconstructing tumor resections. *Clinical Orthopaedics and Related Research*.

[13] Virkus W., Henshaw R. M., Miller B., Gitelis S., Wiesel S. (2010). Use of allografts and segmental prostheses for reconstruction of segmental bone defects. *Operative Techniques in Orthopaedic Surgery*.

[14] Aponte-Tinao L., Farfalli G. L., Ritacco L. E., Ayerza M. A., Muscolo D. L. (2012). Intercalary femur allografts are an acceptable alternative after tumor resection. *Clinical Orthopaedics and Related Research*.

